# Draft genome sequence of *Amycolatopsis camponoti* RTGN1, a bacterial endophyte isolated from *Alnus glutinosa* root nodules

**DOI:** 10.1128/mra.00470-23

**Published:** 2023-12-21

**Authors:** Ryan Michael Thompson, Edward M. Fox, Maria del Carmen Montero-Calasanz

**Affiliations:** 1 School of Natural and Environmental Sciences, Newcastle University, Newcastle upon Tyne, United Kingdom; 2 Department of Applied Sciences, Northumbria University, Newcastle upon Tyne, United Kingdom; 3 IFAPA Las Torres-Andalusian Institute of Agricultural and Fisheries Research and Training, Junta de Andalucía, Cra. Sevilla-Cazalla, Seville, Spain; University of Strathclyde, Glasgow, United Kingdom

**Keywords:** genome, actinobacteria, *Amycolatopsis*, *Alnus glutinosa*, endophyte, nodules

## Abstract

Here, we report the draft genome sequence of *Amycolatopsis* camponoti RTGN1, a bacterial endophyte of *Alnus glutinosa* root nodules, collected from Saltwell Park, United Kingdom. The genome is 11.9 Mbp in size, composed of 147 contigs, with an N_50_ of 179,211 bp and presenting a GC content of 70.9%.

## ANNOUNCEMENT

*Alnus glutinosa* are of interest due to their application in bioremediation (1 – 3). Similarly to legumes, actinorhizal plants present symbiotic root nodules; however, the nodule microbiome remains relatively understudied, with the majority of current knowledge focused upon their principal endosymbiont *Frankiaceae* (4). As such, bacterial isolation was attempted from *A. glutinosa* root nodules to study the non-*Frankiaceae* inhabitants of the root nodules.

*Amycolatopsis camponoti* RTGN1 was isolated from *A. glutinosa* nodules collected from a singular tree in Saltwell Park, Gateshead, United Kingdom (54.944723, –1.605852). Nodules were washed to remove bulk soil, and 4–15 nodule lobes were removed and surface sterilized using 1 mL of 25%-strength household bleach (~1.125% sodium hypochlorite) by agitating the tube for 5 minutes. Sequentially, the bleach was removed and the nodule lobes were washed five times in 1 mL of sterile distilled water for 5 minutes and homogenized in 0.5 mL of 1/4-strength Ringers solution (5). To this homogenate, 14.5 mL of 1/4-strength Ringers solution was added with this plated upon tap water yeast extract agar (50 mg/L nystatin) and incubated at 28°C for 8 days (https://www.dsmz.de/microorganisms/medium/pdf/DSMZ_Medium1625.pdf). Strain RTGN1 was recovered by cultivating a single colony upon tap water yeast extract for 5-7 days at 28°C. Three iterations of subculturing were conducted from a single colony to achieve an axenic culture, with material stored in 30% glycerol at −80°C.

RTGN1 was cultivated upon GYM agar (https://www.dsmz.de/microorganisms/medium/pdf/DSMZ_Medium65.pdf) for 5 days, after which DNA was extracted using a GenElute Bacterial Genomic DNA Kit (Sigma-Aldrich, USA), with an additional ethanol DNA precipitation conducted. This was sequenced by Novogene Co. Ltd., with the DNA library prepared using the Novogene NGS DNA library prep set (Cat No. Pt004) in which the DNA was randomly sheared into short fragments, end repaired, A-tailed, and then ligated with the Illumina adaptor. These sequences were amplified using PCR, and DNA of 350 bp was selected, purified, and sequenced using 150-bp Illumina paired end sequencing upon the Illumina NovaSeq platform.

In the following analysis, standard settings were utilized unless otherwise noted. The 10,687,744 reads generated from sequencing of RTGN1 were filtered using FastP (v0.23.1) (6) to remove adapter sequences, reads that contained more than 10% uncertain nucleotides or more than 50% low-quality reads (base quality < 5). This filtering left 99.7% of the reads, of which 96.4% were deemed Q_20_ and 91.0% were deemed Q_30_. The filtered reads were uploaded to Galaxy Europe (https://usegalaxy.eu/) (7) and assessed using FASTQC (Galaxy Version 0.73+galaxy0) (http://www.bioinformatics.babraham.ac.uk/projects/fastqc/), assembled using Shovill (Galaxy Version 1.1.0+galaxy1) (https://github.com/tseemann/shovill), with contigs quality assessed using Quast (Galaxy Version 5.0.2+galaxy5) (8 – 10). From the assembly, 29 contigs were removed as they were below 200 bp in length. The relationship of RTGN1 to its nearest neighbor, *Amycolatopsis camponoti* A23T, was determined using the TYGS webserver (v389), with 45.2% dDDH similarity noted (11, 12).

The assembled genome was 11,990,019 bp in length, composed of 147 contigs, displaying an N_50_ of 179,211 bp, a GC% content of 70.9%, and an estimated sequencing depth of 134×.

## Data Availability

The draft genome and SRA data of *Amycolatopsis camponoti* RTGN1 have been deposited in INSDC under the accession numbers JAPZLD000000000 and SRR22101359, respectively.
